# The Effect of Non-Immersive Virtual Reality Exergames versus Traditional Physiotherapy in Parkinson’s Disease Older Patients: Preliminary Results from a Randomized-Controlled Trial

**DOI:** 10.3390/ijerph192214818

**Published:** 2022-11-10

**Authors:** Elvira Maranesi, Elisa Casoni, Renato Baldoni, Ilaria Barboni, Nadia Rinaldi, Barbara Tramontana, Giulio Amabili, Marco Benadduci, Federico Barbarossa, Riccardo Luzi, Valentina Di Donna, Pietro Scendoni, Giuseppe Pelliccioni, Fabrizia Lattanzio, Giovanni Renato Riccardi, Roberta Bevilacqua

**Affiliations:** 1 Scientific Direction, IRCCS INRCA, 60124 Ancona, Italy; 2 Clinical Unit of Physical Rehabilitation, IRCCS INRCA, 60127 Ancona, Italy; 3 Clinical Unit of Physical Rehabilitation, IRCCS INRCA, 63900 Fermo, Italy; 4 Medical Direction, IRCCS INRCA, 60127 Ancona, Italy; 5 Neurology Unit, IRCCS INRCA, 60127 Ancona, Italy

**Keywords:** older people, Parkinson’s disease, balance, gait, technology-based intervention, non-immersive virtual reality exergame, virtual reality, exergame, risk of falling, randomized controlled trial

## Abstract

(1) Background: Parkinson’s disease (PD) is one of the most frequent causes of disability among older people. Recently, virtual reality and exergaming have been emerged as promising tools for gait and balance rehabilitation in PD patients. Our purpose is to evaluate an innovative treatment for older patients with PD, based on non-immersive virtual reality exergames, improving gait and balance and reducing falling risk. (2) Methods: Thirty PD patients were recruited and randomly divided into two groups, to receive a traditional rehabilitation (CG) or a technological rehabilitation (TG). (3) Results: A statistical improvement of balance at the end of treatments was observed in both groups (CG: 12.4 ± 0.7 vs. 13.5 ± 0.8, *p* = 0.017; TG: 13.8 ± 0.5 vs. 14.7 ± 0.4, *p* = 0.004), while the overall risk of falling was significantly reduced only in the TG (POMA Total: 24.6 ± 0.9 vs. 25.9 ± 0.7, *p* = 0.010). The results between groups shows that all POMA scores differ in a statistically significant manner in the TG, emphasizing improvement not only in balance but also in gait characteristics (9.7 ± 0.8 vs. 11.4 ± 0.2, *p* = 0.003). Moreover, TG also improves the psychological sphere, measured thorough MSC-(17.1 ± 0.4 vs. 16.5 ± 0.4, *p* = 0.034). Although an improvement in FES-I and Gait Speed can be observed, this increase does not turn out to be significant. (4) Conclusions: Results suggest how non-immersive virtual reality exergaming technology offers the opportunity to effectively train cognitive and physical domains at the same time.

## 1. Introduction

Parkinson’s disease (PD) is a brain disorder that causes unintended or uncontrollable movements, such as shaking, stiffness, and difficulty with balance and coordination [1]. There are currently more than 1.2 million people living with PD in Europe and this number is forecast to double by 2030 [2]. The annual cost per Parkinson’s patient amounts to approximately EUR 11,000 on average across Europe, and a cost to Europe of EUR 13.9 bn annually [3]. There are numerous advantages that may be associated with early therapeutic intervention in PD, such as the decrease of symptoms and the potential for slowing down disease progression, generating a major impact in terms of the quality of life of older patients and the reduction of costs associated with the disease in the long term. Tertiary prevention is an important component of contemporary healthcare for individuals living with PD, as there is growing evidence that exercise and/or physical activity efforts may slow down the decline of functional mobility, while increasing the quality of life [4,5]. Even in the presence of symptomatic relief from medical, surgical, and rehabilitative interventions, in fact, older people with PD face a persistent worsening of disability, characterized by diminished well-being, reduced functional mobility, decreasing performance in activities of daily living, and the worsening of neurological symptoms. Regarding functional decline, guidelines recommend physical therapy early at the onset of the disease [6], but there is not strong evidence on benefits in terms of preventing the beginning of advanced symptoms and the progression of severity, while understanding the key role of gait and balance has important clinical application. Identifying new effective interventions for counteracting disability is a priority in the rehabilitation of PD patients [7,8,9].

At this purpose, recent studies [10,11,12] confirm that technology-delivered balance training may produce performance improvements that are also correlated with evident neurobiological changes in the cerebral cortex [10,11], highlighting the promising role of technological interventions in supporting balance and other motor disorders in PD patients. Virtual reality (VR) technology and exergaming, especially, have been emerged as promising tools for studying and rehabilitating gait and balance impairments in people with PD, as it allows users to be engaged in an enriched and highly individualized complex environment [13]. In particular, exergaming is defined as technology-driven physical activities that requires participants to be active and/or exercise in order to play games, by using the full body motion as a principal mean of interaction [14]. Several studies have shown that interventions based on exergames promote the simultaneous training of cognitive and motor aspects and offer a number of stimuli and difficulty of the tasks adjusted to the patients’ needs, maintaining control and stimulus consistency [15,16,17]. Specifically, the improvement of gait and posture parameters, together with cognitive features, are investigated before and after an exergaming intervention [18,19,20]. In those studies, PD patients improved on balance (i.e., Berg balance score, single leg stands, functional reach test), motor function (i.e., sit to stand, time up and go), the severity of PD motor symptoms (i.e., UPDRS III), and activities of daily living.

Moreover, there is growing evidence that exergames provide a transfer effect from motor to cognitive skills in able-bodied populations, including older adults [21]. More recently, the benefits of exergames on global cognition and individual cognitive domains such as executive functions, attentional processing, and visuo-spatial skills, were demonstrated in both healthy and clinical populations [22].

This study aims to evaluate an innovative rehabilitation treatment for older patients with Parkinson’s disease, based on non-immersive virtual reality exergames, designed to improve gait and balance and to reduce the risk of falling. The treatment involves the use of the Tymo^®^ system (Tyromotion, Graz, Austria), a wireless static and dynamic platform, for evaluating and rehabilitating posture. The primary outcome of the study is the improvement of balance and gait of older PD patients, as a result of the use of technological intervention, at the end of the 10-treatment sessions. Secondly, the impact of the use of technology on the overall quality of life of the participants, is analyzed.

## 2. Materials and Methods

This study represents the preliminary data collected for the clinical trial “Innovative Models in the Rehabilitation of the Elderly with Parkinson’s Disease Through Technological Innovation”, registered on ClinicalTrials.gov with trial registration number NCT04087031 (12 September 2019).

### 2.1. Subejcts

This study is a single blinded (outcome assessors) randomized controlled trial. Participants and physiotherapist are not blinded. Data collection is started in January 2020 and it is ongoing. Thirty-two PD patients were selected by the outpatient department at the Clinical Unit of Physical Rehabilitation, IRCCS INRCA, in the Ancona and Fermo branches, and randomly divided into two groups, to receive a traditional rehabilitation program (CG) or, in addition to the traditional therapy, a technological rehabilitation using Tymo system (TG). A randomization technique based on a single sequence of random assignments is used. A list of random numbers generated by the computer is used and subject is assigned a number based on their order of inclusion in the study. This procedure is conducted by a different researcher from the one who conducted the data analysis. According to this technique, the 32 subjects are randomly assigned to one of the 2 study groups.

Patients were eligible if they were over 65 years old; able to provide informed consent; had a stage of Hoen and Yahr (H&Y) scale between 1 and 3 [23]; had a functional ambulation category (FAC) ≥ 2 [24]; had a ranking scale (RS) score ≤ 3 [25]; had a stability of drug treatment for at least 1 month; negative for geriatric depression scale (GDS) 5-items [26]; had a mini mental state examination (MMSE) ≥ 24 [27]. The evaluation of the compliance with the inclusion/exclusion criteria was performed during the recruitment session. Once we completed this phase, informed consent was obtained and the patients’ assessment was performed at the start and at the end of the treatment. In particular, the baseline evaluation consisted of administration of the following scale: clinical dementia rating scale (CDR) [28], evaluation of the acceptance of the technology with the psychosocial impact of assistive devices scale (PIADS) [29], measurement of functional state with the Barthel index (BI) [30], gait and balance performance on Tinetti’s performance oriented mobility assessment (POMA) [31], evaluation of quality of life with SF-12 health survey (SF-12) [32] and fear of falling with falls efficacy scale-international (FES-I) [33].

### 2.2. Intervention

**Figure 1:**
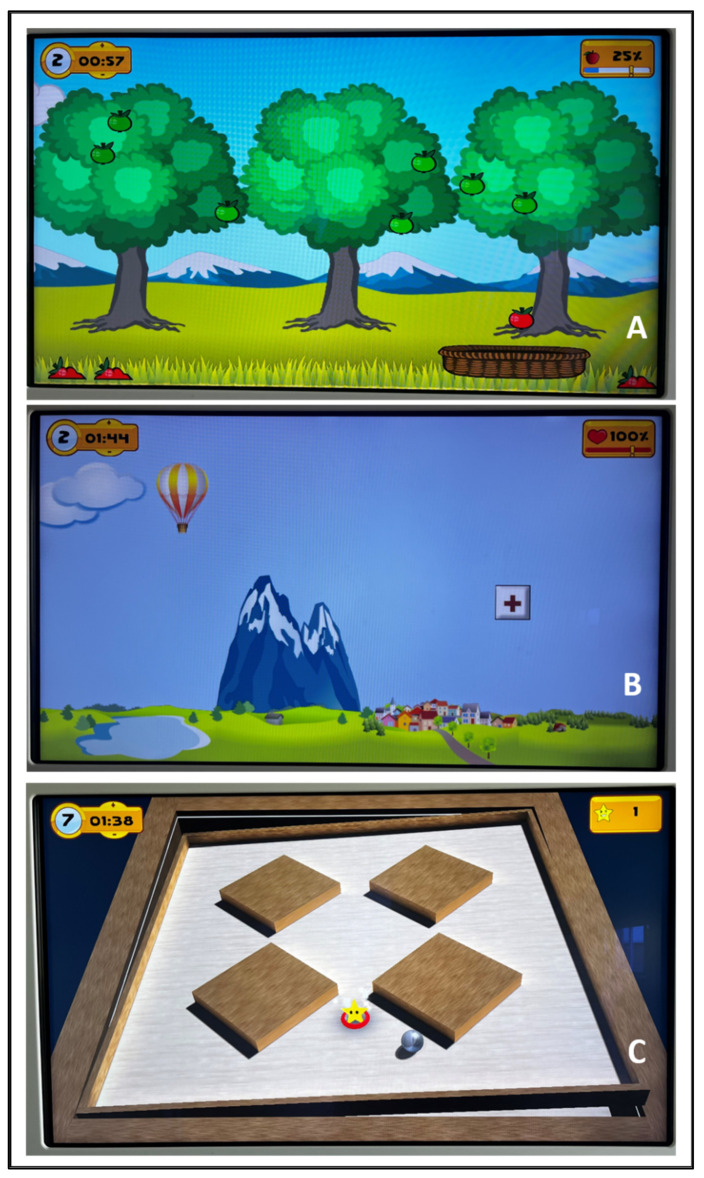
Examples of non-immersive virtual exergame provided by the Tymo^®^ system. (**A**): Non-immersive virtual exergame ‘apple picking’; (**B**): non-immersive virtual exergame ‘the hot-air balloon’; (**C**): non-immersive virtual exergame ‘the labyrinth’.

**Figure 2:**
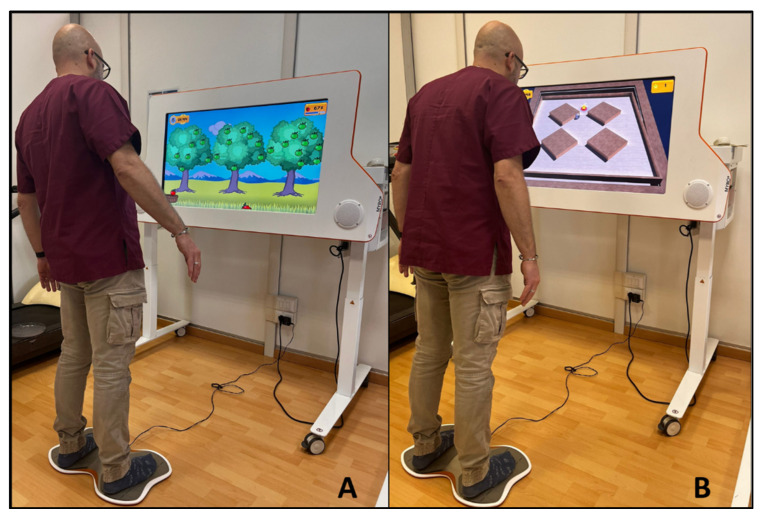
Examples of interaction between subject and the Tymo^®^ system. (**A**): Non-immersive virtual exergame ’apple picking’; (**B**): non-immersive virtual exergame ‘the labyrinth’.

A 10-sessions training was conducted, divided into 2 sessions per week, for 5 weeks. The control group performed traditional therapy sessions lasting 50 min each. The technological intervention group carried out 30 min of traditional therapy and 20 min of treatment with the system [34,35]. All patients included in the study perform traditional rehabilitation treatments, consisting in breathing and relaxation; task-oriented exercise to improve strength and to reduce limitations in the activities of daily living; walking with cues to reduce gait deficit; stretching to relieve muscle and joint stiffness; static and dynamic balance training to reduce postural control impairments; flexibility exercises to improve the range of motion of different joint; unilateral and contralateral coordination exercises performed in bed and standing involving the 4 limbs. The technological treatment consists of using the Tymo^®^ system. It is a wireless platform that provides non-immersive virtual reality exergames, which can be adapted to each patient according to the functional capacity, in order to improve balance and postural control (Figure 1). The patient is placed on the platform in front of a screen where the non-immersive virtual exergames are shown. For their execution, the patient’s body becomes the joystick that, moving in space, reaches the different targets of the game (Figure 2). Through the non-immersive virtual reality exergames proposed by the system, the physiotherapist can decide to work in one dimension (antero-posterior or latero-lateral) or in two dimensions (combining antero-posterior and latero-lateral movements). In particular, the system offers a number of therapy games from Verena Schweizer’s neurotraining. For example, a type of one-dimensions exergame is ‘apple picking’: the patient, moving the center of gravity sideways, controls the movement of a basket to pick up the apple that is falling from the tree. The falling speed of apple and the number of apples on the tree can be set by the physiotherapist according to the patient’s characteristics (Figure 1A). The apples collected and the time taken to complete the game are counted towards the next level. Another typical exergame is ‘the hot-air balloon’: the patient controls the movement of the hot-air balloon to avoid obstacles such as mountains or clouds. In this case, the hot-air balloon speed and the number of obstacles can be set (Figure 1B). An example of two-dimensions exergame is ‘the labyrinth’: the objective is to move a ball on a plane with obstacles so that it reaches a precise point on the plane highlighted by a star. In this case, the patient, moving in all directions, controls the movement of the plane by sliding the ball across it (Figure 1C).

In this way, the intervention involves not only the physical domain but also the cognitive.

### 2.3. Outcomes

All outcome measures follow a standardized operating procedure. In particular, the primary outcome of the study is the improvement of balance, gait and the fear of falling of older PD patients, measured through the three POMA scale (POMA balance, POMA gait and POMA total), as a result of the use of the technological intervention, at the end of the 10-treatment sessions. Secondly, the gait speed of older PD patients, the fear of falling (FES-I), the level of autonomy in daily living activities (BI) and the physical and psychological state of the patients (SF-12) are analyzed.

### 2.4. Statistical Analysis

Descriptive data were presented as mean and standard deviation (SD) for continuous variables or numbers (percentage) for categorical ones. Since we had a small sample size, determining the distribution of the variables was important for choosing the most appropriate statistical method. In line with this, Shapiro-Wilk test was performed and did not show evidence of non-normality. Based on this, we decided to use a parametric test. Additionally, the mean and standard deviation were used to summarize the variables reported. Pearson’s chi-squared test for categorical variables and Student’s *t* test for continuous variables were applied to test statistically significant differences (*p* < 0.05) between CG and TG parameter mean values. Before/after comparison was assessed with matched-pairs Student’s *t* test. The statistical analysis was performed using the SPSS software.

## 3. Results

**Figure 3:**
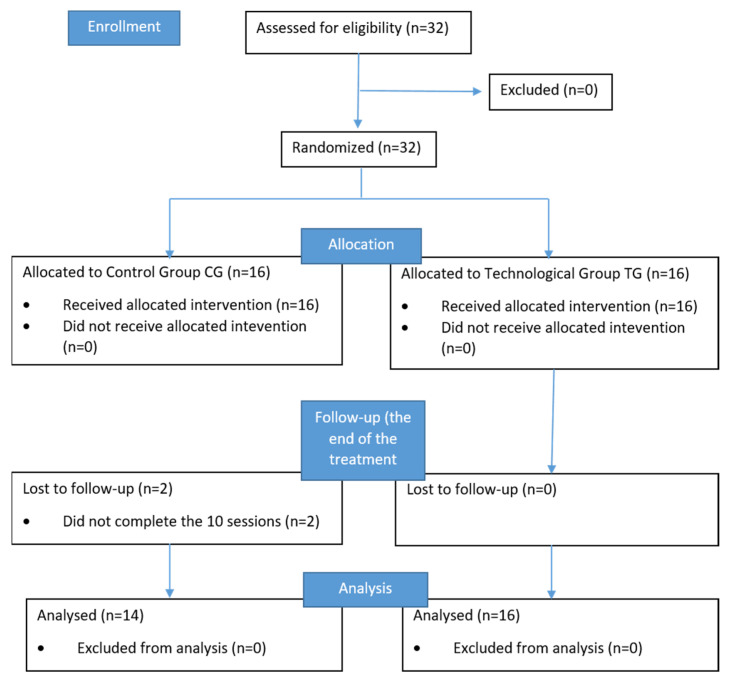
The CONSORT flowchart.

Demographic, clinical and functional data of the sample (14 subjects for CG and 16 subjects for TG) are reported in Table 1. Two participants in the CG dropped out because they did not complete the treatment. The CONSORT (consolidated standards of reporting trials) flowchart is shown in Figure 3.

At the baseline, no differences in the inclusion criteria value (Hoehn and Yahr score; ranking scale score; geriatric depression scale; functional ambulation category; mini mental state examination) and in the demographic characteristics (gender; age; marital status; educational level) were found between the two groups, emphasizing the homogeneity of the two groups.

Table 2 shows pre- and post-intervention scores and differences between groups at the start and the end of the treatment of each group on the functional state scales with the Barthel index (BI), gait and balance performance on Tinetti’s performance oriented mobility assessment (POMA gait and POMA balance), evaluation of the quality of life with SF-12 health survey (SF-12) and its sub-scores (physical component score PCS-12 and mental component score MCS-12), fear of falling (FES-I), together with the gait speed.

Statistical analysis reveals a significant effect on POMA balance performance after intervention in both groups. Moreover, in the TG, the overall execution of POMA total has improved. Although an improvement in FES-I and gait speed can be observed, this increase does not turn out to be significant. In particular, in the CG, both the falls efficacy scale and the gait speed show a slight increase, whereas, in the TG, the values of these two variables remain almost unchanged.

The comparison of the two groups shows that the improvement in balance is greater in the group using the platform than in the control group at the end of the treatment. In addition, the assessment of gait and fall risk at the end of the treatment, measured with POMA gait and POMA total, respectively, shows a statistically significant difference between the two groups, underlining the advantage of using the technology for the rehabilitation of PD patients. Another relevant result is the improvement (*p* = 0.034) of the mental component score of the SF-12 scale (MCS-12) in the TG respect to CG, highlighting the efficacy of the non-immersive virtual reality exergames also in the emotional and mental health status.

## 4. Discussion

This study was designed to investigate the effect of a technological intervention based on non-immersive virtual reality exergames on gait, balance and fear of falling in patients with Parkinson’s disease, performed with the Tymo^®^ platform. Technological rehabilitation based on exergaming may represent a novel and more effective exercise model, compared to the traditional approach, as it integrates physical and cognitive exercises in an interactive digital, augmented or virtual game-like environment. [36].

In line with this, our results confirmed the beneficial effects of technological intervention over the standard therapy, as demonstrated by the assessment of the primary outcome, the POMA scale. In fact, as a statistical improvement of balance, (POMA balance scale), has been observed in both groups, while the overall risk of falling (POMA total score) [37] has been significantly reduced only in the experimental group. Furthermore, the literature suggests that the rehabilitative program for PD should be “goal-based” (targeted to practicing and learning specific activities in the core areas), with a number of practice variables (intensity, specificity, complexity) that need to be personalized to the individual patients’ characteristics [38], as in the case of Tymo^®^ system. Moreover, exergames seem to increase synaptic strength and influences neurotransmission, thus potentiating functional circuitry in PD [39]. In fact, exercise interventions in individuals with PD incorporate goal-based motor skill training in order to engage cognitive circuitry important in motor learning. Using this exercise approach, physical therapy facilitates learning through instruction and feedback (reinforcement), and encouragement to perform beyond self-perceived capability.

A deeper analysis of the results between groups shows that all three POMA scales’ scores (balance, gait and total) differ in a statistically significant manner, emphasizing the improvement not only in balance but also in gait characteristics in the experimental group. These results suggest that a standard therapy combined with an innovative treatment using Tymo^®^ is more effective for training of physical performance in PD patients. It can be hypothesized that this kind of platform allows to train the patient static balance together with the dynamic, managing, for example, the improvement in knee extension, step height and gait security. Moreover, to maintain balance during physical exercise, the patient does rely on both feedback and feedforward control. Furthermore, the patient has to simultaneously perform a visual exploration activity, activating visual and dual tasking cognitive control [40]. The lack of significance in the other scores may be due to a ceiling effect, given that many of the subjects reached the upper limit that was set for the scale.

In addition to the enhancement at functional level, our results show a statistically significant evaluation of the psychological sphere: the mental-component scale (MCS) of SF-12 has been improved in the experimental group, that have performed the technological intervention [41]. As described by Ware et al. [42], the MCS focuses on emotional status such as depression, anxiety and carelessness. As a combination of exercises and interactive features, the technological intervention provided through the Tymo^®^ platform seemed to positively influence the psychological well-being of the older participants.

Recently, a systematic review has underlined the capability of exergames to protect the psychological status of older people from worsening [43] and thus remaining cognitively healthy. In line with the results of other authors, our findings suggest a positive effect on mood after technological intervention, that can thus be considered as a complementary tool for rehabilitating older adults with PD, thanks to the high degree of acceptability of the games. As physical activity is an essential part of therapy for PD patients, engaging approaches may have the pivotal role of increasing adherence as much as possible, by including also therapeutic ingredients to counteract the onset of depression and cognitive decline. Effective technological-base rehabilitation that is easily adapted for patients with PD could be used as a supplement or alternative to conventional therapy. Moreover, this type of training has the advantage of involving patients to increase adherence to therapy in the long term [44,45], assuring a higher engagement of the PD patient in the rehabilitation path [46,47].

Despite the positive results collected, we acknowledge that this study has several limitations that should be considered in light of the results. First of all, a higher number of participants would be beneficial for the generalization of the findings. Moreover, additional follow-up measurements would be relevant to understand if the improvement in the selected variables is sustained over time. Finally, longer follow-up would allow the inclusion of the history of falls as potential outcome for future studies.

Nevertheless, our study is important to encourage the diffusion and use of innovative rehabilitative approaches for PD, that includes a combination of standard therapy with advanced technological solutions, like exergames, to also provide a positive impact on the psychological status, in addition to functional mobility and the overall quality of life.

## 5. Conclusions

This pilot study represents a starting point in the use of technology in the rehabilitation of the patient with Parkinson’s disease. In fact, our results suggest how non-immersive virtual reality exergaming technology offers the opportunity to effectively train different domains at the same time, such as cognitive and physical domains, highlighting the potential role in the rehabilitation settings thanks to the scalability and personalization of the intervention.

## Figures and Tables

**Table 1:** Baseline demographic and clinical profile.

	CG n = 14	TG n = 16	*p*
Gender, n (%)			0.153
Female	5 (35.8%)	10 (62.5)	
Male	9 (64.2%)	6 (37.5%)	
Age, mean ± SD	75.5 ± 5.4	72.7 ± 6.3	0.131
Marital status, n (%)			0.891
Married	12 (85.7%)	14 (87.5%)	
Widowed	2 (14.3%)	2 (12.5%)	
Educational level, n (%)			0.204
Primary education	5 (35.7%)	10 (62.5%)	
Secondary education	7 (50%)	4 (25%)	
University or more	2 (14.3%)	2 (12.5%)	
Hoehn and Yahr score, mean ± SD	2.3 ± 0.9	2.0 ± 0.8	0.187
Rankin scale score, mean ± SD	1.6 ± 0.7	1.2 ± 0.9	0.201
GDS, mean ± SD	2.5 ± 1.2	2.5 ± 1.4	0.987
FAC, mean ± SD	4.1 ± 1.2	4.6 ± 2.4	0.052
MMSE, mean ± SD	26.6 ± 1.9	27.0 ± 1.8	0.295

CG = control group; TG = technological group; SD = standard deviation; GDS = geriatric depression scale; FAC = functional ambulation category; MMSE = mini mental state examination.

**Table 2:** Mean ± standard error of the mean of pre- and post-intervention scores on the BI, POMA (total, gait and balance), SF-12 (total, physical and mental component score), FES-I and gait speed. Pre-post and between groups comparisons are reported for each score (*p* < 0.005).

	CG		*p*-Value	TG		*p*-Value	*p*-Value CG vs. TG	
	T0	T1		T0	T1		T0	T1
BI	90.3 ± 3.9	87.6 ± 4.5	0.251	91.88 ± 2.4	94.3 ± 3.7	0.281	0.687	0.367
POMA								
POMA Total	22.2 ± 1.2	23.3 ± 1.6	0.208	24.6 ± 0.9	25.9 ± 0.7	0.010 **^**	0.142	0.034 *
POMA Gait	9.7 ± 0.5	9.7 ± 0.8	0.905	10.9 ± 0.4		0.185	0.352	0.003 *
POMA Balance	12.4 ± 0.7	13.5 ± 0.8	0.017 **^**	13.8 ± 0.5	14.7 ± 0.4	0.004 **^**	0.249	0.034 *
SF-12						11.4 ± 0.2		
SF-12-Tot	30.3 ± 0.7	30.3 ± 0.7	0.651	31.6 ± 0.7	30.1 ± 0.6	0.055	0.476	0.094
PCS-12	13.2 ± 0.6	13.2 ± 0.5	0.856	13.8 ± 0.4	13.6 ± 0.3	0.786	0.303	0.953
MCS-12	17.1 ± 0.4	17.1 ± 0.4	0.692	17.8 ± 0.5	16.5 ± 0.4	0.022 **^**	0.750	0.034 *
FES-I	12.1 ± 1.6	14.1 ± 1.8	0.750	13.9 ± 1.1	13.3 ± 1.4	0.898	0.425	0.312
Gait Speed [m/s]	1.6 ± 0.8	1.7 ± 0.9	0.140	1.8 ± 0.7	1.8 ± 0.1	0.472	0.623	0.350

CG = control group; TG = technological group; T0 = baseline; T1 = end of the treatment; BI = Barthel index, POMA gait = Tinetti’s performance oriented mobility assessment-gait part; POMA balance = Tinetti’s performance oriented mobility assessment-balance part, SF-12 = SF-12 health survey; SF-12-Tot = SF-12 health survey total score; PCS-12 = SF-12 physical component score; MCS-12 = SF-12 mental component score; FES-I = falls efficacy scale—international; * *p*-values Student’s *t* test; ^ *p*-values from matched-pairs Student’s *t* test.

## Data Availability

The datasets generated, used and analyzed during the trial and its preceding pilot trial are or will be available from the corresponding author upon reasonable request.
